# 4581 Autonomic Dysfunction as a Marker of Depression and Coronary Artery Disease

**DOI:** 10.1017/cts.2020.291

**Published:** 2020-07-29

**Authors:** Anish Sanjay Shah

**Affiliations:** 1 Emory University

## Abstract

OBJECTIVES/GOALS: Dysfunction of the autonomic nervous system (ANS) may be important in both depression and coronary artery disease (CAD). A novel heart rate variability (HRV) metric, *Dyx*, may be a be a potentially useful tool to study ANS dysfunction in these diseases. We propose that ANS dysfunction, measured by decreased *Dyx*, will associate with both depression and obstructive CAD. METHODS/STUDY POPULATION: We included participants undergoing coronary angiography for suspected CAD. Depressive symptoms were assessed with the Patient Health Questionnaire-9 (PHQ-9). HRV data were collected continuously on participants before catheterization using a new ECG patch (VivaLNK). We assessed HRV by *Dyx* (primary) and high and low frequency power, multiscale entropy, and deceleration capacity. Two-sample t-tests and logistic regressions (with adjustment for age and sex) were used to study the difference in HRV (before cardiac catheterization) between those with high versus low depressive burden (PHQ-9 ≥ 10), and in those with versus without obstructive CAD (>70% stenosis). RESULTS/ANTICIPATED RESULTS: We assessed 30 individuals with mean (SD) age 62.4 (13.2); 7.1% were female and 15.4% were black. Mean *Dyx* in high depressive symptoms (N = 21, 70%) was 1.8 (0.2) and in none-low depressive symptoms (N = 7, 23%) was 2.2 (0.2). Differences were also observed for high frequency (HF) (4.4 (1.1) vs. 6.0 (1.4)) and deceleration capacity (−4.2 (2.1) vs. −10.7 (8.5)). Mean *Dyx* in obstructive CAD (N = 17, 57%) and non-obstructive CAD (N = 10, 33%) was 1.7 (0.6) and 2.6 (1.2) respectively. Differences were seen with sample entropy (1.2 (0.2) vs. 1.5 (0.2)). Every 1 unit of log(HF) had an odds ratio = 0.14 (95% CI 0.06 – 0.36) for depression. DISCUSSION/SIGNIFICANCE OF IMPACT: ANS dysfunction, measured by HRV, associates with both depression and obstructive CAD. Autonomic ECG markers may play an important role in assessing brain-heart pathology, and may be useful to study the interaction between depression and CAD.

